# Association between antibody-mediated immune responses and gastroesophageal reflux disease: Evidence from genetic studies

**DOI:** 10.1097/MD.0000000000043254

**Published:** 2025-07-11

**Authors:** Liqun Li, Lijian Liu, JinJing Tan, Hongxia Pan, Chengning Yang, Jieru Xie, Jing Yan, Jinchan Peng, Xiaoyan Huang, Sheng Xie

**Affiliations:** a The First Affiliated Hospital of Guangxi University of Chinese Medicine, Nanning, Guangxi, China; b Graduate School of Guangxi University of Chinese Medicine, Nanning, Guangxi, China.

**Keywords:** antibody-mediated immune responses, gastroesophageal reflux disease, inflammatory factors, mediating analysis, Mendelian randomization

## Abstract

Studies have proven an association between the specific antibody-mediated immune response and gastroesophageal reflux disease (GERD); however, the exact causal associations remain unclear. Our research aims to evaluate the causal relationships between the genetic susceptibility to 46 antibody-mediated immune responses and GERD, with the bidirectional two-sample Mendelian randomization (TSMR) and to explore the mediating effects of 91 circulating inflammatory cytokines by a two-step MR analysis. Multiple MR analysis methods, including inverse variance weighted, MR-Egger, weighted mode, weighted median, simple mode, and MR pleiotropy residual sum and outlier, were employed for the bidirectional TSMR analysis. Besides, sensitivity analyses, such as Cochran Q statistics, MR-Egger intercept, MR pleiotropy residual sum and outlier global test, and the leave-one-out method were implemented to identify potential heterogeneity and pleiotropy. In the forward MR analysis, the inverse variance weighted method demonstrated that anti-cytomegalovirus (anti-CMV) IgG seropositivity (OR = 1.031, 95% CI: 1.000–1.062, *P* = .048), levels of human herpes virus (HHV) 7 U14 antibody (OR = 1.097, 95% CI: 1.032–1.167, *P* = .003), and herpes simplex virus 1 (HSV-1) mgG-1 antibody (OR = 1.069, 95% CI: 1.020–1.119, *P* = .005), which have higher genetic prediction, are positively related to GERD risks. Reverse MR analysis revealed GERD increased the level of Epstein–Barr virus VCA p18 antibody (OR = 1.255, 95% CI: 1.028–1.533, *P* = .026), while decreased the anti-CMV IgG seropositivity (OR = 0.663, 95% CI: 0.444–0.991, *P* = .045) and HHV 7 U14 antibody level (OR = 0.777, 95% CI: 0.630–0.958, *P* = .018) as well. Besides, other antibodies, including anti-*Helicobacter pylori* (*H pylori*) IgG, *H pylori* CagA, and *H pylori* VacA demonstrated no correlations (neither positive nor negative) with GERD risks. Surprisingly, TSMR showed that 91 cytokines seemed not to mediate HSV-1 mgG-1 to enhance GERD risks. The research provided convincing evidence for establishing a causal relationship between antibody-mediated immune responses and GERD, proving that genetic-prediction-based HSV-1 mgG-1 could increase GERD risk and that inflammatory factors may not be involved in mediating this association. Besides, GERD was found to reduce Epstein–Barr virus VCA p18 antibody. Moreover, we revealed the bidirectional causal relationships between GERD and the antibodies, including anti-CMV IgG, HHV 7 U14. This research may contribute to a better understanding of the mechanism that triggers GERD, also emphasizing the clinical potential of therapeutic interventions targeting antibody-mediated immune responses.

## 1. Introduction

Gastroesophageal reflux disease (GERD) encompasses a range of discomforts resulting from the backflow of stomach contents into the esophagus.^[1]^ This disease can be classified as non-erosive reflux disease, erosive esophagitis, and Barrett esophagitis. The clinical manifestations of GERD are multifaceted, mainly characterized by acid reflux and heartburn, often accompanied by extra-esophagus symptoms such as chronic cough, hoarseness, laryngitis, and palpitations.^[1]^ Evidence derived from meta-analysis has revealed that GERD now presents a global morbidity of 13.98%, with male morbidity of 15.69% and 17.17% in females. In terms of racial groups, 12.92% of Asian populations suffer from GERD, while in the European populations it is 14.12%, in Latin American and Caribbean populations 12.8%, and in North American populations 19.55%.^[2]^ Over the past 2 decades, the total number of cases and deaths of GERD have increased by 74.79% and 77.19%, respectively.^[3]^ Clinical treatments of GERD mainly focus on suppressing gastric acid and promoting gastric motility. Proton pump inhibitors serve as first-line medications, effectively mitigating mucosal damage and reflux symptoms. However, refractory GERD persists in approximately 40% of patients after an 8-week double-dose proton pump inhibitors treatment.^[4]^ Besides, plenty of research has demonstrated a significant association between GERD and multiple other diseases, exemplified by the aggravation of chronic obstructive pulmonary disease and esophageal adenocarcinoma, which may cause a further negative impact on the patients, heavier economic burden, and bring a more critical challenge to clinical treatments as well.^[5]^ Therefore, deepening the understanding of GERD risk factors and pathogenic mechanisms may facilitate the development of targeted therapies.

The exact pathogenesis of GERD remains unclear. By far, various studies have indicated that the main pathological mechanisms of GERD may involve esophageal mucosa damage due to decreased function of esophageal clearance, anti-reflux barrier mechanisms, and gastroesophageal motility disorder.^[6]^ Besides, in recent years, research has shown another main factor in the onset of GERD, which is the gastroesophageal microecological imbalance.^[7]^ Antibodies are proteins produced by the body in response to the stimulation from antigens. It is the core of the anti-pathogenic reaction of the adaptive immune system in mammals. As the excavation of the pathological mechanism of GERD deepens, studies have discovered that the antibody-induced immune reactions may play a crucial role in the pathological progress of GERD. To exemplify, levels of anti-cytomegalovirus (anti-CMV), produced when the body is infected by human CMV, are independently related to the increasing epithelial intestinal damage and translocation of microorganism,^[8]^ also related to multiple dyspeptic outcomes.^[9]^ Meanwhile, studies have shown that anti-CMV IgG levels increase the levels of inflammatory factors, such as C-X-C motif chemokine 13, IL-6, IL-8, and TNF-α.^[8]^ Imbalance of factor response mechanisms is a key pathologic mechanism in GERD. So far, multiple studies have discussed the causal relationships between GERD and antibody-mediated immune responses, e.g., *Helicobacter pylori* (*H pylori*) related antibodies. Clinical research showed that *H pylori* plays a protective role on GERD via the development of gastric atrophy.^[10]^ The level of *H pylori* antibody (e.g., vacAS1, cagA) demonstrates a negative correlation with GERD, presenting a potential protective effect against the disease.^[11–13]^ However, contradicting conclusions from other research indicated that *H pylori* is one of the causes of GERD,^[14]^ and the level of vacA, one of its antibodies, is positively associated with GERD.^[15]^ Moreover, latest research showed no evidence of associations between *H pylori* infections and GERD.^[16]^ Therefore, causal relationships between immune responses mediated by specific antibodies and GERD remain unclear. This conflict arises because all the evidence above comes from observational research, which can be influenced by confounding factors, reverse causality, and other biases. Clinically, it is important to design comprehensive and accurate research to evaluate the relationships between antibody-mediated immune responses and GERD risks for optimizing prevention strategies and reducing morbidity and clinical damage.

Mendelian randomization (MR) research utilizes genetic variations to explore how modifiable exposures influence causality in different results and is regarded as a “natural” randomized controlled trial.^[17,18]^ Based on Mendel laws of inheritance and instrumental variable (IV) estimation method, MR is effective in mitigating confounding factors in traditional observative epidemiological research, and the bidirectional two-sample MR (TSMR) is employed to address possible reverse causality problems.^[18]^ By far, thanks to rapid identification and the complex character-associated genetic varieties in the genome-wide association studies (GWAS), MR has become a popular epidemiological method for inferring the causal relationships between the disease outcomes and the exposure.^[19]^ Based on previous studies, we posed the scientific question for this study: what is the causal relationship between predicted antibody-mediated immune responses and GERD risk? If a causal relationship exists, is it mediated by inflammatory factors? Besides, there has been a lack of TSMR-applied research exploring these relationships. Therefore, this research aims to reveal potential causal relationships between antibody-mediated immune response and GERD risks via TSMR, as well as to reveal whether inflammatory factors are mediators of the antibody immune response affecting GERD via two-step MR analysis, thereby providing strategies for GERD prevention and treatments.

## 2. Methods

### 2.1. Study design and assumption

**Figure 1.**
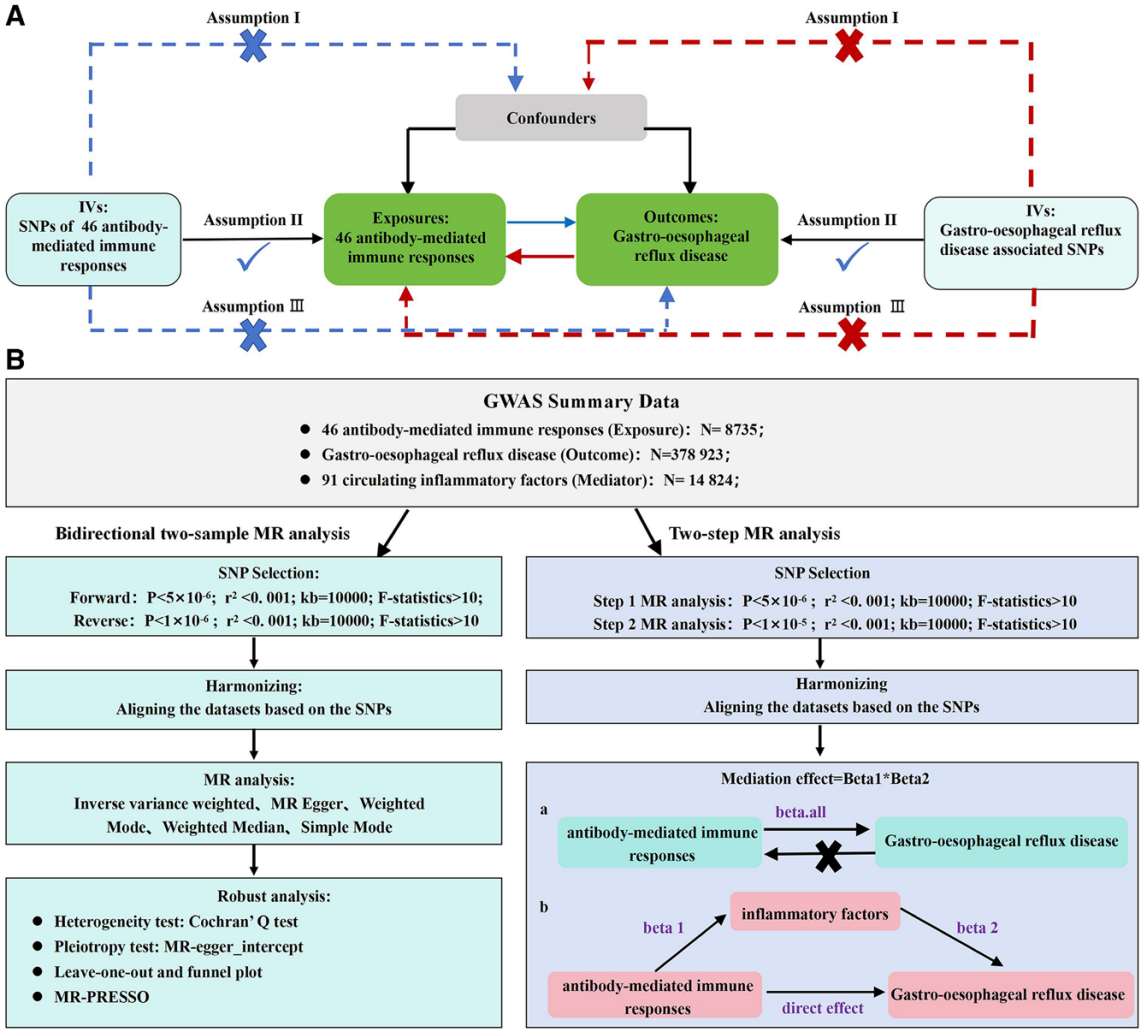
(A) Schematic diagram of Mendelian randomization principles. (B) Flow chart of two-way two-sample MR analysis.

The study applied TSMR analysis to evaluate causal relationships between antibody-mediated immune responses and GERD. Figure 1 shows the summary of the study design. Data were drawn from publicly available statistics from previous publications or collaborative datasets. Therefore, no permissions are required for this research. There are 3 compulsory assumptions for MR analysis.^[20]^ (i) Independence: applied genetic variants should have no associations with any confounding factors that may influence the relationship between antibody-mediated immune response and GERD. (ii) Relevance: applied genetic variants should have strong relevance with the exposure. (iii) Exclusion restriction: the genetic variants can only influence outcome through the exposure. First, the genetic variants of 46 antibody-mediated immune responses were selected to explore their causal relationships with GERD. Second, specific causal links between GERD and each antibody-mediated immune response were established based on the selected genetic variants. Finally, TSMR was applied to implement a mediated analysis on 91 circulating inflammatory cytokines to deduce the mediating role that they play in the causal relationships. Quality control steps for summary data of GWAS were performed in this research to select associated single nucleotide polymorphism (SNP), and the research report follows the latest guideline of Strengthening the Reporting of Observational Studies in Epidemiology-Mendelian randomization.^[21]^

### 2.2. GWAS data sources for antibody-mediated immune responses

In MR analysis, it is critical to select appropriate genetic variants. All the cases and controls were selected among European populations to solve biases due to racial confounding factors. Noticeably, every sample cohort is independent of each other. All the GWAS data about antibody-mediated immune responses applied in the MR analysis in this research came from a published GWAS research including 8735 European individuals. This research utilized genome-wide genotyping of infectious diseases in the UK Biobank cohort, including 46 phenotypes of antibody immunoreactivity that were defined by data from 13 pathogens. Detailed elucidation of data management can be found in the original publication.^[22]^

### 2.3. GWAS data sources for GERD

Launched in 2017 in Finland, the FinnGen database program is one of the largest genome research programs aimed at enhancing human health via genetic research to uncover new therapeutic targets and diagnostic methods for multiple diseases.^[23]^ The GWAS summary data of GERD come from FinnGen (ID: finngen_R10_K11_REFLUX.gz). The research involved 378,923 participants, among which comprising 28,859 GERD cases and 350,064 controls from the European population. Since all participants were Europeans and there was no overlap of samples, the racial bias was avoided.

### 2.4. GWAS data sources for inflammatory cytokines

Data of 91 circulating inflammatory cytokines came from 11 cohorts of GWAS that measured with Olink Target Inflammation Panel, involving overall 14,824 participants from the European individuals.^[24]^ More detailed information of data management can be found in the original publication.^[24]^

### 2.5. Genetic instruments

To ensure a strong correlation with the exposure, we chose IVs with the specificity of genome-wide significance (*P* < 5e-6) from the antibody-mediated immune responses dataset, from the GERD dataset with genome-wide significance (*P* < 1e-6) and from the inflammatory factors dataset with genome-wide significance (*P* < 1e-5). To test the independence and linkage disequilibrium effects of these variables, the parameters for removal of linkage disequilibrium effects were set to clump kb = 10000, clump *r*^2^ = 0.001. Besides, we used the F-statistic to assess the strength and precision of the IV-exposure association. F > 10 indicates a strong association and precision able to avoid biases induced by weak IVs. Characteristics of involved SNPs are listed in Tables S1 to S3, Supplemental Digital Content, https://links.lww.com/MD/P357.

### 2.6. Bidirectional TSMR analysis

A bidirectional TSMR analysis was conducted in this research to evaluate the causal relationships between antibody-mediated immune responses and GERD. First, TSMR was utilized as a forward analysis to examine the causal relationships between genetic-predicted antibody-mediated immune responses and GERD. Afterward, genetic instruments of GERD were selected as IV, and antibody-mediated immune responses as the outcome of in a reversed MR analysis, the causal effect of GERD genetic variation on the antibody-mediated immune response was then determined. As the inverse variance weighted (IVW) method is regarded as one of the most effective analyzing methods,^[25,26]^ MR predictive values were obtained with IVW meta-analysis. Besides, MR-Egger,^[27]^ weighted mode,^[28]^ weighted median,^[29]^ simple mode, and MR pleiotropy residual sum and outlier (MR-PRESSO) were also involved in the research for secondary analysis.

For sensitivity analysis, pleiotropic identification and calibration were implemented via the MR-Egger intercept test.^[30]^ The causal estimates from MR-Egger regression slope were used to correct for unbalanced pleiotropy, and to determine the pleiotropic effects (*P* < .05). IV Funnel plots, IVW, Cochran Q test, and MR-Egger were also conducted to assess SNP heterogenicity, with *P* > .05 indicating a less possibility of heterogenicity.^[25,31]^ MR-PRESSO was also utilized to evaluate potential pleiotropy. The confirmed outlier would be eliminated before a new analysis was performed to identify and calibrate the horizontal pleiotropy.^[32]^ Leave-one-out (LOO) sensitivity analysis was conducted to identify any single SNP that drove the causal association. IVW method, was implemented after SNP elimination, to evaluate the effectiveness of the rest SNPs to the outcome, thereby inferring the stability of effect size. In this case, *P* < .05 indicated a significant association. All MR analyses were performed with the “Two Sample MR” and the “Mendelian Randomization” package in the R software (Version R 4.3.3).

### 2.7. Mediation analysis

Furthermore, mediation analysis was implemented via TSMR to explore the mediating effect of 91 circulating inflammatory cytokines in the causal relationship between antibody-mediated immune responses and GERD. First, the MR analysis of antibody-mediated immune responses and inflammation cytokines was performed to obtain Beta1; then Beta2 was gathered via MR of inflammation cytokines and GERD. MR of antibody-mediated immune response and GERD was performed in the third step to gain Beta. all. Finally, the mediation effect analysis was determined via the 2 formulas: mediation effect = Beta1 × Beta2; direct effect = Beta. all - Beta1 × 2 (Fig. 1).

## 3. Results

### 3.1. Genetic instruments selection

For the forward MR, a series of filtrations were performed on the SNPs of antibody-mediated immune responses based on the 3 assumptions of MR. The afterward SNPs characteristics of 46 antibody-mediated immune responses were listed in Table S1, Supplemental Digital Content, https://links.lww.com/MD/P357. All the selected SNPs were robust instruments with F-statistics ranging from 20.776 to 343.063, and *R*^2^ from 0.002 to 0.102. Quantities of each antibody-mediated immune response located ranged from 5 to 59, with *P* < 5e-6.

For the reversed MR, GERD was applied as the exposure to perform its causal influence on the antibody-mediated immune responses. Following the same procedure, 9 SNPs were picked from the summary data of the European population group as IVs (Table S2, Supplemental Digital Content, https://links.lww.com/MD/P357). All the selected IVs were not weak nor did any linkage disequilibrium, instead, strong IVs (*P* < 1e-6), and their F-statistics ranged from 23.98 to 31.00.

For the mediation analysis, the inflammatory cytokines was applied as the mediators. Quantities of each inflammatory cytokine located ranged from 5 to 44, with *P* < 1e-5. All selected SNPs were robust instruments with F-statistics ranging from 19.51 to 3549.33 (Table S3, Supplemental Digital Content, https://links.lww.com/MD/P357).

### 3.2. Forward MR analysis

**Figure 2.**
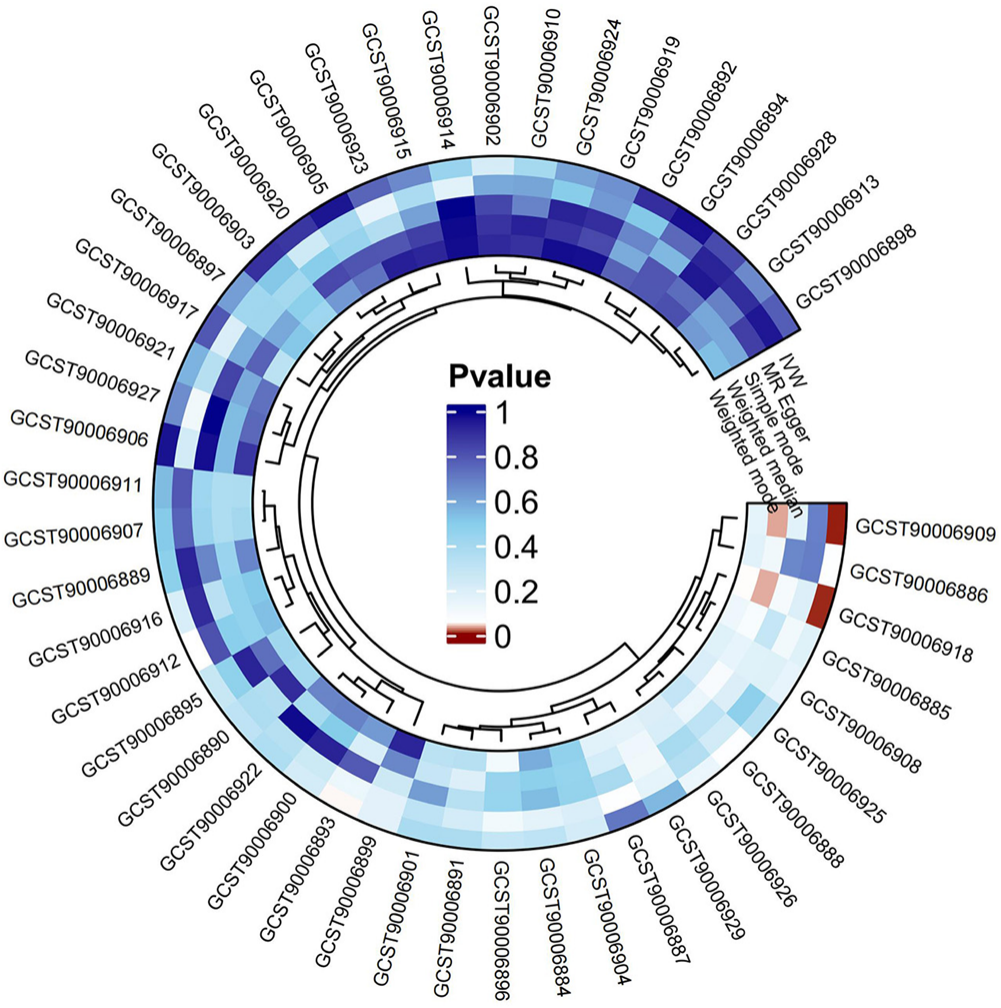
Circos heatmap of the causal relationships between 46 antibody-mediated immune responses and GERD as observed in the forward MR analysis. GERD = gastroesophageal reflux disease, MR = Mendelian randomization.

**Figure 3.**
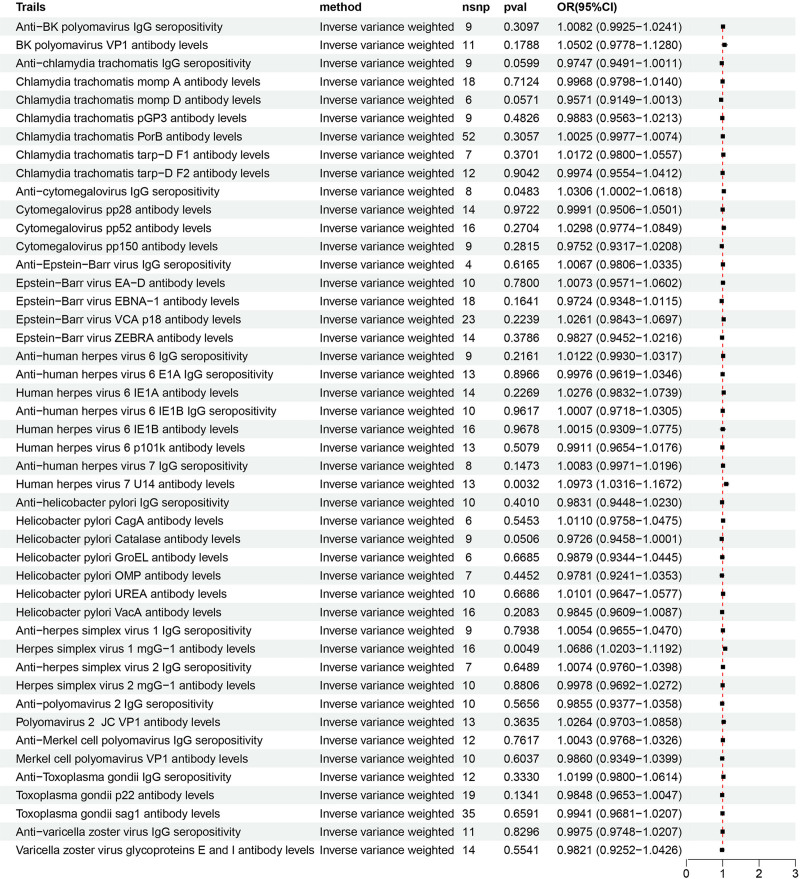
Forest plots of the causal relationships between 46 antibody-mediated immune responses and GERD as observed in the forward MR analysis. GERD = gastroesophageal reflux disease, MR = Mendelian randomization.

In the forward MR analysis, Table S4, Supplemental Digital Content, https://links.lww.com/MD/P357 demonstrated detailed MR outcomes of different methods. Forward MR outcomes indicated that the genetic susceptibility of 3 antibody-mediated immune responses associated and GERD risk have a positive causal relationship, while that of 43 other antibody-mediated immune responses including *H pylori* IgG, *H pylori* CagA, *H pylori* GroEL, and *H pylori* VacA showed no evidence of significant association with GERD risks (Fig. 2). Sensitivity analysis indicated that all the estimated values were not heterogeneous and pleiotropic (Table S5, Supplemental Digital Content, https://links.lww.com/MD/P357). Specifically, the primary result of IVW revealed that anti-CMV IgG seropositivity (OR = 1.031, 95% CI: 1.000–1.062, *P* = .048), levels of human herpes virus (HHV) 7 U14 antibody (OR = 1.097, 95% CI: 1.032–1.167, *P* = .003), and herpes simplex virus 1 (HSV-1) mgG-1 antibody (OR = 1.069, 95% CI: 1.020–1.119, *P* = .005) were strongly associated with the increase of GERD risk (Fig. 3). The MR-PRESSO method further confirmed the reliability of these causal relationships (Table S6, Supplemental Digital Content, https://links.lww.com/MD/P357). Besides, the estimated value provided by the weighed median also proved the positive casual relationships between HHV 7 U14 antibody levels, HSV-1 mgG-1 antibody levels and GERD risks (Table S4, Supplemental Digital Content, https://links.lww.com/MD/P357).

**Figure 4.**
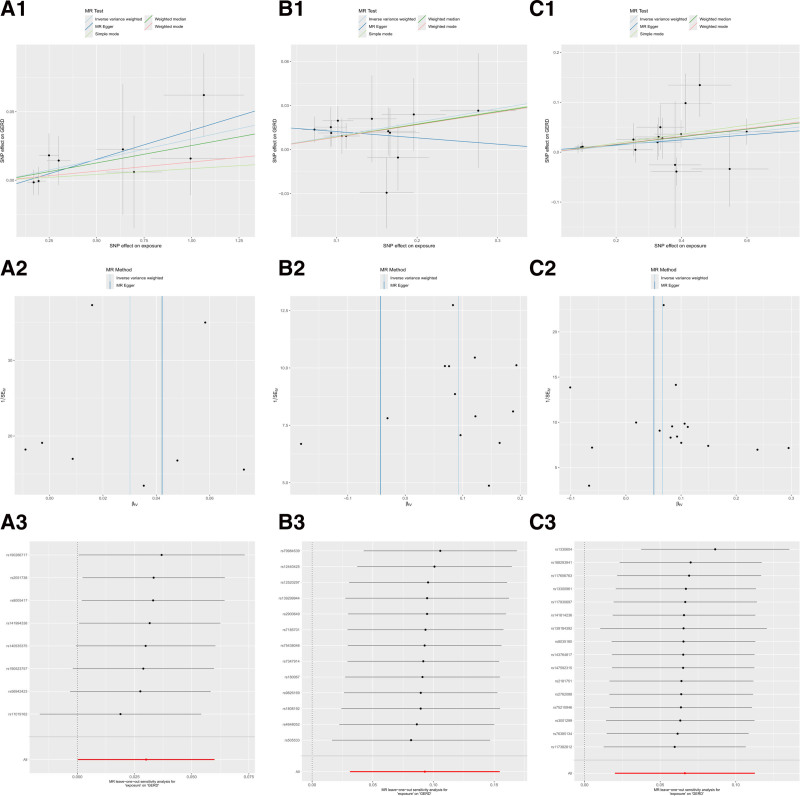
Scatter plots (1), funnel plot (2), and LOO analysis (3) for causal effects of antibody-mediated immune responses related traits on GERD (forward). (A) Anti-cytomegalovirus IgG seropositivity. (B) Human herpes virus 7 U14 antibody levels. (C) Herpes simplex virus 1 mgG-1 antibody levels. GERD = gastroesophageal reflux disease, LOO = leave-one-out.

Directional pleiotropy does not affect the MR-Egger regression intercept test of in TSMR analysis regarding the effect of genetic susceptibility to the immune response of the above 3 antibodies on GERD risk (Table S5, Supplemental Digital Content, https://links.lww.com/MD/P357). Scatter plots of the different methods visualized the anti-CMV IgG seropositivity, levels HHV 7 U14 antibody, and HSV-1 mgG-1 antibody were strongly associated with the increase of GERD risk (Fig. 4A1–C1). Consistent results were also obtained with MR-PRESSO (Table S5, Supplemental Digital Content, https://links.lww.com/MD/P357). Besides, the outcomes of the Cochran Q test that applied IVW and MR-Egger methods did not demonstrated heterogeneity among the selected SNPs (Table S5, Supplemental Digital Content, https://links.lww.com/MD/P357 and Fig. 4A2–C2). Similarly, the results of the LOO sensitivity analysis demonstrated that the outcomes of MR analysis were not driven by a single SNP (Fig. 4A3–C3). Overall, the results of the sensitivity analysis manifested the robustness of MR analysis.

### 3.3. Reverse MR analysis

**Figure 5.**
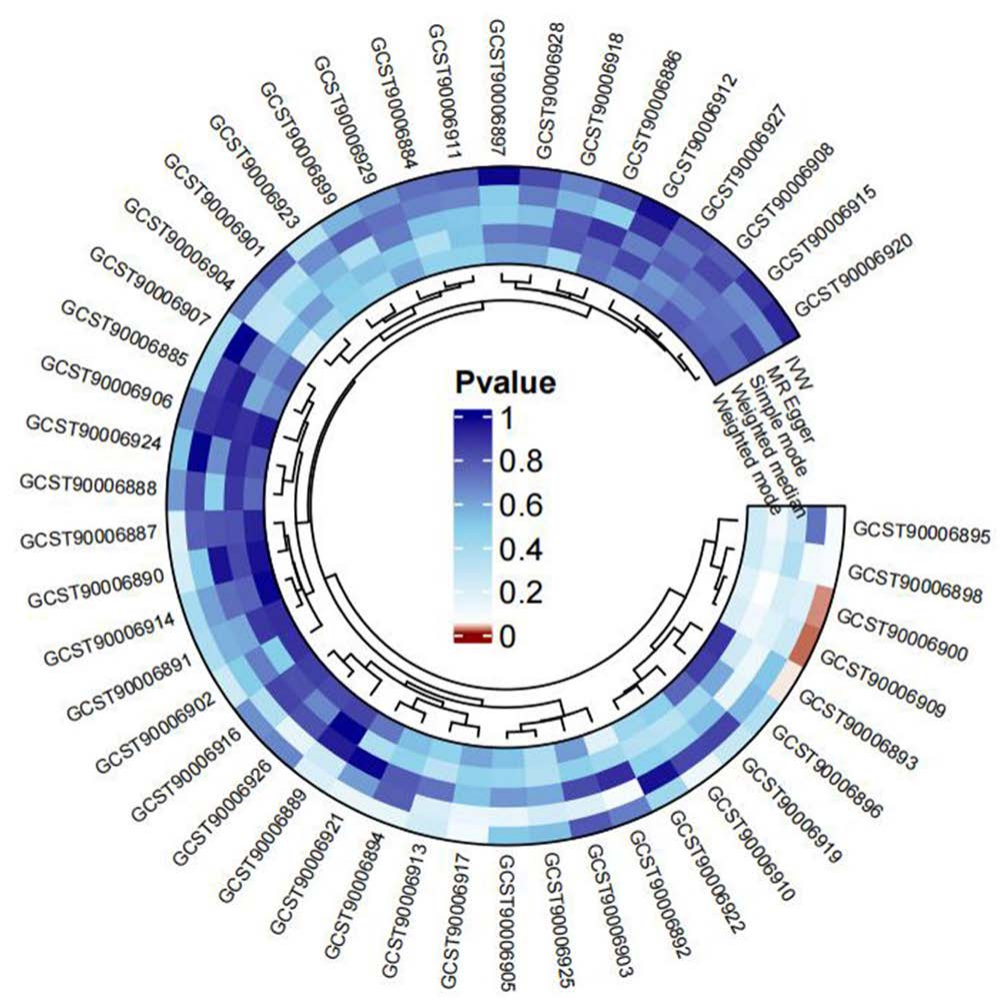
Circos heatmap of the causal association between 46 antibody-mediated immune responses and GERD as observed in the reverse MR analysis. GERD = gastroesophageal reflux disease, MR = Mendelian randomization.

**Figure 6.**
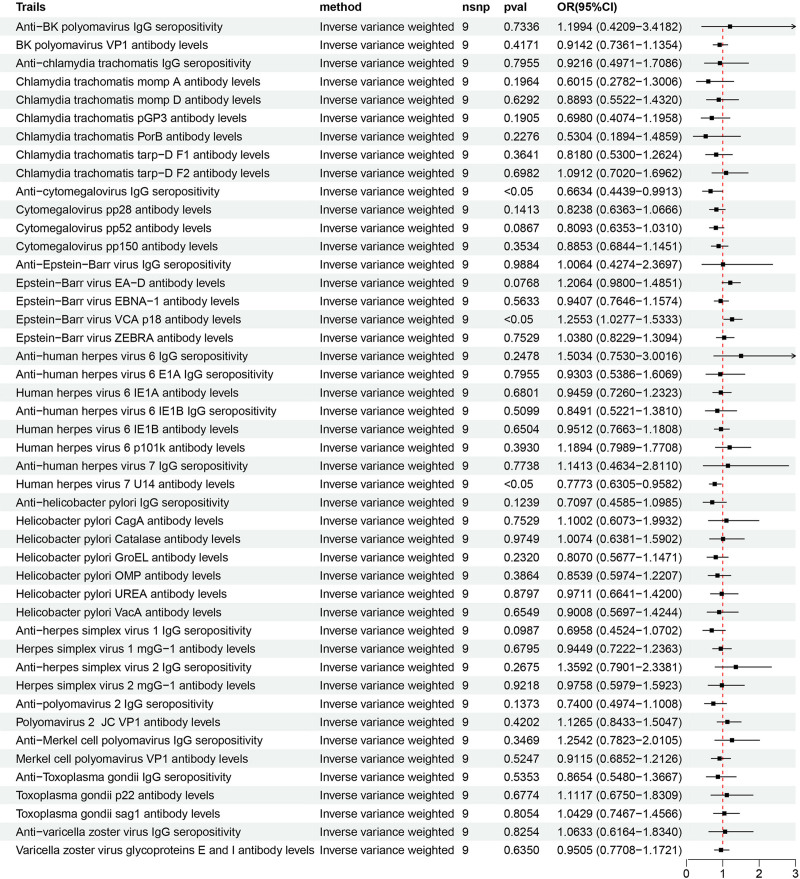
Forest plots of the causal association between 46 antibody-mediated immune responses and GERD as observed in the reverse MR analysis. GERD = gastroesophageal reflux disease, MR = Mendelian randomization.

Reverse MR analysis was applied to explore the causal relationships between the genetic susceptibility to GERD and the antibody-mediated immune responses. Detailed outcomes of different MR methods are listed in Table S7, Supplemental Digital Content, https://links.lww.com/MD/P357. The reverse MR analysis indicated a significant causal correlation between GERD and genetic susceptibility to 3 antibody-mediated immune responses, while no substantial relevance was detected between 43 other antibody-mediated immune responses, including *H pylori* IgG, *H pylori* CagA, *H pylori* GroEL, and *H pylori* VacA (Fig. 5). The sensitivity analysis demonstrated no hint of heterogeneity among all the SNPs, nor pleiotropy in estimated values (Table S8, Supplemental Digital Content, https://links.lww.com/MD/P357). IVW outcomes showed a positive correlation between GERD and Epstein–Barr virus (EBV) VCA p18 antibody levels (OR = 1.255, 95% CI: 1.028–1.533, *P* = .026), while it is a negative correlation was observed with anti-CMV IgG seropositivity (OR = 0.663, 95% CI: 0.444–0.991, *P* = .045) and HHV 7 U14 antibody levels (OR = 0.777, 95% CI: 0.630–0.958, *P* = .018) (Fig. 6). In the MR-PRESSO analysis, despite the determined causal relationships between EBV VCA p18 antibody and GERD, estimated values of the association between GERD risks and the genetic susceptibility to the other 2 antibody-mediated immune responses showed no substantial change (Table S9, Supplemental Digital Content, https://links.lww.com/MD/P357).

**Figure 7.**
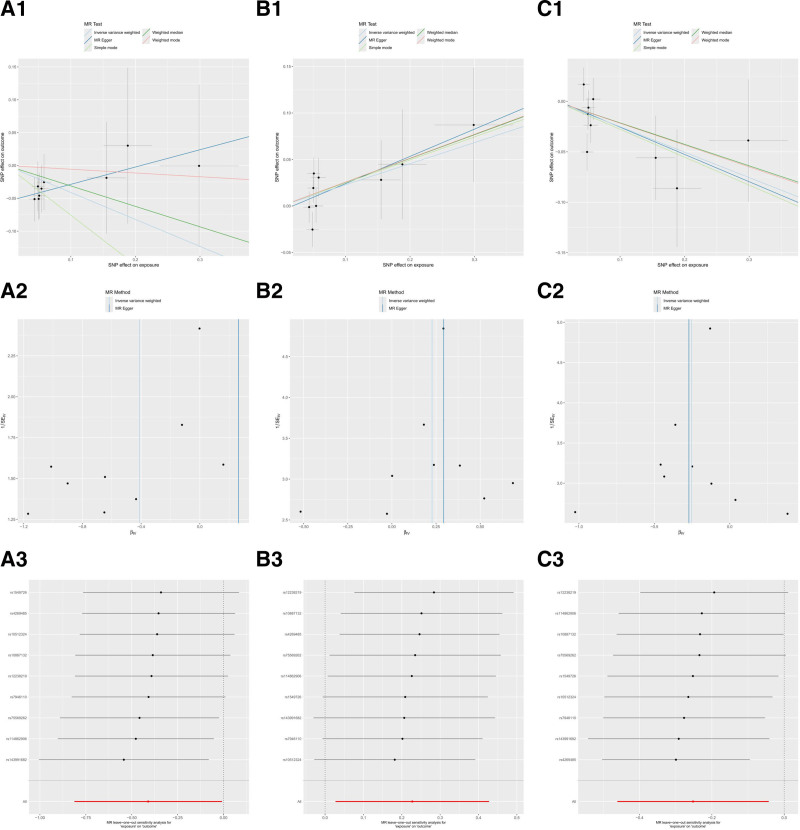
Scatter plots (1), funnel plot (2), and LOO analysis (3) for causal effects of GERD-related traits on antibody-mediated immune responses (reverse). (A) Anti-cytomegalovirus IgG seropositivity. (B) Human herpes virus 7 U14 antibody levels. (C) Herpes simplex virus 1 mgG-1 antibody levels. GERD = gastroesophageal reflux disease, LOO = leave-one-out.

MR-Egger intercept results indicated that there was no pleiotropy in the causal relationships between the 3 antibody-mediated immune responses and GERD (Table S9, Supplemental Digital Content, https://links.lww.com/MD/P357). Scatter plots from the IVW method visualized that EBV VCA p18 antibody level positively was associated with GERD risk, while anti-CMV IgG seropositivity and HHV 7 U14 antibody level showed a significant negative correlation (Fig. 7A1–C1). Besides, the Cochran Q test after the IVW and MR-Egger methods showed no heterogeneity among selected SNPs (Fig. 7A2–C2). Similarly, the LOO sensitivity analysis pointed out that biases caused by few SNPs with heterogeneity were relatively small (Fig. 7A3–C3). Overall, the results of the sensitivity analysis manifested the robustness of MR.

### 3.4. Mediation analysis

According to the TSMR results above, mediation analysis was added to investigate whether the 91 circulating inflammatory cytokines mediate the relationship between HSV-1 mgG-1 and increased risk of GERD. The results showed a negative correlation between HSV-1 mgG-1 and C-X-C motif chemokine 10 (OR = 0.940, 95% CI: 0.887–0.997, *P* = .039), while positive correlation between HSV-1 mgG-1 and fibroblast growth factor 19 (OR = 1.070, 95% CI: 1.009–1.135, *P* = .024) (Table S10, Supplemental Digital Content, https://links.lww.com/MD/P357). However, no significant causal relationship between these inflammatory cytokines and GERD was established (Table S11, Supplemental Digital Content, https://links.lww.com/MD/P357). Besides, C-X-C motif chemokine 1 (OR = 1.052, 95% CI: 1.004–1.103, *P* = .035), interleukin-1-alpha (OR = 1.083, 95% CI: 1.018–1.152, *P* = .012), and interleukin-4 (OR = 1.081, 95% CI: 1.018–1.147, *P* = .011) were positively related to GERD, and the tumor necrosis factor ligand superfamily member 12 (OR = 0.958, 95% CI: 0.917–1.000, *P* = .049) was negatively correlated Table S11, Supplemental Digital Content, https://links.lww.com/MD/P357). However, no significant causal relationship was found between HSV-1 mgG-1 and these inflammatory cytokines (Table S10, Supplemental Digital Content, https://links.lww.com/MD/P357). Pleiotropy was not found in the statistics mentioned above (Tables S10 and S11, Supplemental Digital Content, https://links.lww.com/MD/P357). Generally, there was no evidence indicating that the inflammatory cytokines mediate the relationship between HSV-1 mgG-1 and the onset of GERD.

## 4. Discussion

Plenty of previous research has reported causal relationships between immune responses mediated by certain antibodies (e.g., *H pylori*-related antibodies IgG, CagA, GroEL, and VacA) and GERD; however, controversy remains intense. As far as it is concerned, this research stands out as the first to comprehensively collect GWAS data of antibody-mediated immune response and applied TSMR on them alongside GWAS data of GERD, thereby evaluating causal relationships between 46 antibody-mediated immune responses and GERD risks. A substantial discovery was announced, demonstrating the role of anti-CMV IgG seropositivity, HSV-1 mgG-1 level, and HHV 7 U14 antibody level in increasing GERD risks. It was also confirmed that GERD increased the level of EBV VCA p18 antibody, and decreased the anti-CMV IgG seropositivity and HHV 7 U14 antibody level. While *H pylori*-related antibody IgG, CagA, and VacA showed no evidence of causally correlated with GERD. Besides, the inflammatory cytokines were found not to mediate the relationship between HSV-1 mgG-1 and the increased GRED risk. Noticeably, the robustness of the outcomes above was supported by sensitivity analysis. To conclude, the results of this research provided promising elucidation to the role of antibody-mediated immune responses in the management of GERD.

As a kind of herpesviruses, the human CMV could be found all over the world. Research reported that CMV could be detected in the upper gastrointestinal mucosa of 23.8% of patients with mucosal ulcers or defect.^[33]^ In large-scale epidemiological research, the increasing level of anti-CMV immunoglobulin G (anti-CMV IgG) is related to adverse health outcomes.^[9]^ Primary infection of CMV mainly affects mucosal epithelial cells, including those in the gastrointestinal tracts.^[34]^ It has been proven that CMV is able to replicate actively in intestinal cells, resulting in the loss of intestinal barriers integrity.^[35]^ Mucosal damage is jointly caused by gastric juice-induced direct damage and immune-inflammatory mechanisms, to be more specific, inflammatory cytokines released by esophageal mucosal epithelium may lead to the migration of neutrophils, thereby causing chronic inflammation.^[6]^ The latest research indicated that anti-CMV IgG levels independently correlated with increased intestinal epithelial damage and translocation of microorganisms, leading to elevated levels of CSCL13, IL-6, IL-8, and TNF-α.^[8]^ Pre-clinical evidence showed a significant increase in IL-6, IL-1β, and IL-8 levels in a rat model of acute reflux esophagitis.^[36]^ Clinical evidence revealed the association between inflammatory factors IL-1β, IL-8 and the histomorphology changes of esophageal mucosa.^[37]^ As the severity of the disease rises, the level of plasma IL-1β, IL-8, and tumor necrosis factor ligand superfamily member 12-α in GERD patients may gradually increase.^[38]^ The research above has indicated that anti-CMV IgG level may increase GERD risks, providing indirect support to this research results, while further confirmation is still required. Therefore, anti-CMV IgG can be regarded as a promising target for GERD treatment, and a deep elucidation of its mucosa-regulating function may provide a potential approach for targeted GERD treatments.

Human herpes virus 7 (HHV 7) is a recently discovered universal β-herpesvirus that may infect immune cells.^[39]^ Phosphoprotein 85, aka U14, can be used as a target for the human immune response to HHV 7, as it could be detected in HHV 7 affected cells and is regarded as one of the main determinants of immune response to HHV 7.^[40,41]^ The immune dominant protein in HHV 7 is the production of the U14 gene.^[40]^ Therefore, HHV 7, whether independently or combined with other β-herpesvirus, could be an important precipitating factor for individuals with immunosuppression developed into severe diseases.^[40]^ Chronic inflammation caused by gastroesophageal motility disorder-induced reflux of gastric contents into the esophagus is considered one of the main mechanisms of GERD. Research found that the elevation of TNF-α, TGF-β, and IFN-γ levels in the supernatant of HHV 7-infected cells demonstrates the significant immune regulating effects of HHV 7 infection on the cytokine’s synthesis and inhabitation of lymphocyte proliferation via multiple stimulations.^[39]^ Compared to normal esophageal tissues, the homing of CD3 + and CD4 + cells were detected increasing in biopsied ex-vivo cultures of GERD. and was expressed into CD103 + via tissue TGF-β.^[42]^ It has been discovered that the morbidity of primary dyskinesia in patients with CD4-dominated esophagitis is significantly higher than that in patients with CD8-dominated esophagitis.^[43]^ A hypothesis was then proposed that the evidence above may indirectly support the discovery of this research, which is that HHV 7 U14 may be the risk factor for GERD.

The research found that HSV-1 mgG-1 increases the risk of GERD. Herpes simplex virus 1 (HSV-1), a well-developed human pathogen, demonstrated an infection rate as high as 67% among the 0 to 49 age group.^[44]^ Mainly infecting oral mucosal epithelial cells, HSV-1 may spread from the original infected sites to the cell bodies of sensory neurons located within the trigeminal ganglion, thereby establishing lifelong latent infections.^[45]^ HSV-1 may infect human gastrointestinal tracts via oral secretions acting as carriers or via neuron transportation. It is considered one of the major causes of esophagitis in hosts with low- or normal-immune function.^[45,46]^ Research reported that HSV-1 was detected in 9.5% of patients with esophageal mucosal ulcers or defects.^[33]^ The evidence above provided support for the conclusion of this research. From the perspective of mechanism, the mediation analysis applied in this research indicated that inflammatory cytokines do not play a mediating role in the increased risk of GERD by HSV-1 mgG-1. After the depletion of CD8 + cells by treatment of specific monoclonal antibodies, intestinal motility disorder of HSV-1 infected mice witnessed partial improvement.^[47]^ After infecting the central nerve system, the neurotropic virus may even reach the enteric nervous system (ENS) and survive. The ENS is a network of nerves embedded within intestine walls. HSV-1 infection leads to continuous expression of virus antigen in intestinal neurons, resulting in elevated activated lymphocytes CD3 + and CD8+. Responses of T cells on HSV-1 antigens that are expressed continuously in the intestinal neurons may change ENS integrity, thereby causing neuromuscular dysfunction.^[47]^ Therefore, it is inferred that the positive correlation between HSV-1 mgG-1 and GERD risk may be due to the gastroesophageal motility disorder caused by HSV-1-induced gastroesophageal ENS damage.

EBV is confirmed as the first human oncogenic virus that is able to establish lifelong asymptomatic persistence. It has been estimated that about 90% of the world population is infected by EBV with established latency in lymphocytes.^[48,49]^ Associated with multiple autoinflammatory diseases, including systemic lupus erythematosus, multiple sclerosis, rheumatoid arthritis and multiple lymphatic malignancies, such as Sjogren syndrome, Hemophagocytic Lymph histiocytosis, as well as carcinoma,^[49–52]^ EBV is considered crucial in triggering autoimmune and autoinflammatory diseases.^[49]^ It has been reported that EBV was detected in upper gastrointestinal mucosa in 38.1% of patients with mucosal ulcers or defects.^[33]^ EBV is not only often re-activated, but also armed with multiple mechanisms to escape from the immune system of the host. Besides, it is also involved in inducing the immune dysfunction of the host, which can trigger or worsen the inflammation process. Research revealed that the increasing level of antibodies indicates the intermittent reactivation of EBV.^[53]^ In the reverse MR performed in this research, GERD was detected a significant positive correlation with the increased level of EBV VCA p18. This may be explained by inflammatory mediators produced largely in the process of GERD, exemplified as IL-6 and IL-8,^[54,55]^ which may induce repeated immune activation; other possible explanations may be the increasing antibody level caused by the reactivation of latent virus, or EBV reaction due to the weakened immune system of GERD patients, or even the increased vulnerability of GERD-effected individuals to EBV. Besides, the reverse MR outcomes also indicated that GERD decreased the genetic susceptibility to anti-CMV IgG and HHV 7 U14, which may result from that GERD patients are more vulnerable to CMV and HHV 7 and are not easier to get fully infected. The findings above supposed that the detection of EBV, CMV, and HHV 7 is necessary in GERD patients to exclude the damage induced by relevant infections and antibody-mediated immune responses.

With a global infection rate of 43.1%,^[56]^ *H pylori* may induce expression of relevant antibodies, e.g., IgG, vacA, CagA, in the body of the host. Clinical research from Japan proposed that *H pylori* may provide a protective effect on reflux esophagitis by causing gastric atrophy.^[10]^ Clinical observational research showed that vacA S1 and cagA, both *H pylori* antibodies, may reduce GERD risks.^[11–13]^ Another observational research demonstrated that *H pylori* was considered one of the causes of GERD,^[14]^ more specifically, its antibody vacA s2 was detected with a positive correlation to GERD.^[15]^ However, since the research mentioned above was all observational, they could be easily affected by confounding factors or reverse casualties, thereby causing controversy. Besides, a previous TSMR analysis proposed a significant positive association between *H pylori* IgG level and GERD risk.^[57]^ However, since the research is a single-way TSMR, the reverse causality was not taken into consideration. In contrast, our bidirectional TSMR analysis, addressing confounding factors and reverse causalities, found no causal relationships between GERD risks and *H pylori*-related antibodies, including IgG, Catalase, GroEL, OMP, UREA, CagA, and VacA. Besides, the research identified and excluded heterogeneity and pleiotropy via rigorous sensitivity analysis, thereby strengthening the reliability of the results. Due to the biofunctions of these antibody-mediated immune responses and the current bottleneck of GERD prevention and treatments, large-scale, multicenter clinical research is necessary to further confirm the causal relationships.

There are multiple strengths of this research. (i) Regarding this research, it represents the first attempt to explore the causal relationships between 46 antibody-mediated immune responses and GERD risk at a genetic-prediction level. This research confirmed the genetic-prediction-based HSV-1 mgG-1 may enhance the GERD risks, and GERD may lower the level of EBV VCA p18 antibodies; bidirectional causal relationships lie between GERD and antibodies, including anti-CMV IgG, HHV 7 U14. (ii) To the best of our knowledge, this study is also the first two-step MR based on the largest GWAS data to explore the potential mediating effect of 91 inflammatory factors in the causal relationship between some specific antibody-mediated immune response and the risk of sepsis and sepsis (28-day mortality), confirming that inflammatory factors may not be mediators of these causal relationships. (iii) Compared to observational research, MR analysis is relatively not susceptible to unknown confounding factors or reverse causalities. Widely applied in confirming the causal relationships between exposures and outcomes, although, randomized controlled trials often require a large amount of time and economic expense. The major advantage of bidirectional TSMR lies in less impact from confounding factors, reverse causality, and non-differential exposure. Meanwhile, we conducted a sensitivity analysis using several different methods to ensure consistent robustness of the results. These findings provide promising guidance to further research and clinical approaches to disease prevention and treatment.

The further directions of this research are: (i) currently, this research mainly focuses on European populations; data from other populations may also be taken into future consideration to improve the universality and applicability of the outcomes. (ii) Although the bidirectional causal relationships between GERD and antibodies, including HSV-1 mgG-1, EBV VCA p18, anti-CMV IgG, and HHV 7 U14 was established based on genetic prediction, detailed biological and genetic mechanisms of the relationships remain unclear. Further research may consider deepening relevant investigations to provide more effective interference for GERD patients. (iii) Other latent confounding factors that may influence the association between antibody-mediated immune responses and GERD, including social and economic status, educational levels, and living environments. These factors should be considered in further research to achieve a more comprehensive understanding of these 2 conditions. (iv) Since TSMR methods applied in this research have provided genetic-prediction-based evidence, further randomized controlled trials research is suggested to confirm the exact causal relationships between GERD and these antibody-mediated immune responses with positive outcomes. Meanwhile, prospective studies with large sample sizes may also be launched to observe the evolution of these relationships over time and their variations across different age and lifestyle groups. (v) Based on the findings of this research, further research of targeted intervention on the antibody inflammatory responses above and GERD may be implemented to evaluate the possible intervention on the prognosis of GERD patients.

The limitations of this research are listed as follows: (i) the data focused on the European individuals, which may restrict the generalizability of the findings to other Ethnic groups. (ii) Despite genetic factors, other factors that may also affect the relationship between antibody-mediated immune responses and GERD, such as environment and lifestyle, were not taken into consideration. (iii) Due to the application of GWAS summary data, it is not possible to involve stratified analysis in this research based on parameters such as age and gender.

## 5. Conclusions

Generally, this research confirmed the increase of GERD risk due to genetic-prediction-based anti-CMV IgG seropositivity, HSV-1 mgG-1 level, and HHV 7 U14 antibody level. GERD was found to increase the level of EBV VCA p18 antibody, and decrease the anti-CMV IgG seropositivity and HHV 7 U14 antibody level. There was not any causal relationship between GERD and *H pylori*-related antibodies, such as IgG, CagA, and VacA. The discoveries of this research may provide a better understanding of GERD mechanisms and also emphasize the potential of intervention treatments targeted at antibody-mediated immune responses.

## Acknowledgments

We are grateful to the investigators who publicized the GWAS data included in this study. Special thanks to Dr Yun Wen for guiding our project.

## Author contributions

**Conceptualization:** Sheng Xie.

**Funding acquisition:** Lijian Liu, Sheng Xie.

**Methodology:** Xiaoyan Huang.

**Project administration:** Sheng Xie.

**Software:** Liqun Li, Chengning Yang.

**Visualization:** Lijian Liu, Hongxia Pan, Jinchan Peng.

**Writing – original draft:** Liqun Li, Jinjing Tan, Xiaoyan Huang.

**Writing – review & editing:** Jieru Xie, Jing Yan, Sheng Xie.

## Supplementary Material


